# Retention of Tritiated Alcohols by Zeolites Used in Indoor Air Sampling

**DOI:** 10.1021/acsomega.6c08364

**Published:** 2026-09-24

**Authors:** Lukas Zellinger, Dominik Boya, Veronika Rosecker, Bin Feng, Georg Steinhauser

**Affiliations:** † Institute of Applied Synthetic Chemistry & TRIGA Center Atominstitut, 27259 Technische Universität Wien, Vienna 1060, Austria; ‡ Institute of Radiation Medicine, 12478 Fudan University, Shanghai 200437, China

## Abstract

Zeolite 4 Å, typically used in air samplers for indoor tritium monitoring, was tested for the uptake of organically bound tritium (OBT) compounds, in particular low-molecular-weight alcohol vapors. In a straightforward synthesis, tritiated methanol (CH_3_OT, **1**) and ethanol (CH_2_CH_2_OT, **2**) were obtained from the hydrolysis of the respective alkoxides with tritiated water (aqueous HTO solution). In an independent approach, methyl-group tritiated methanol (CH_2_TOH, **3**) was produced via hydrolysis of [^3^H]­methyl-4-nitrobenzenesulfonate. Blank experiments for the tritium exchange on hydrated glass surfaces were conducted. Tritium losses were found negligible under ambient conditions. These tritiated alcohols were applied in batch experiments to test the sorption of their vapor onto zeolite 4 Å. After 24 h of exposure of the zeolite to the alcohol vapor, sorbed tritium compounds were desorbed by leaching with H_2_O and quantified by liquid scintillation counting. At room temperature (22 °C), the sorption of tritiated alcohol vapor by the zeolite (as quantified via the recovery of activity in the leachate) is nearly quantitative. At elevated temperature (60 °C), the activity recovery decreases down to 62–75%. By contrast, the zeolite loading of sorbed alcohols (as expressed by mass of sorbed alcohol per mass of zeolite) increases with the same temperature increase from 20.3 ± 2.0 mg g^–1^ to 30.9 ± 2.8 mg g^–1^ for **1**; from 10.7 ± 1.0 mg g^–1^ to 31.0 ± 2.6 mg g^–1^ for **2**; and from 12.1 ± 2.1 mg g^–1^ to 34.7 ± 2.0 mg g^–1^ for **3**. Under the simulated environmental conditions, zeolite 4 Å shows sufficient affinity toward dilute tritiated methanol and ethanol to be considered useful for indoor air monitoring.

## Graphical abstract

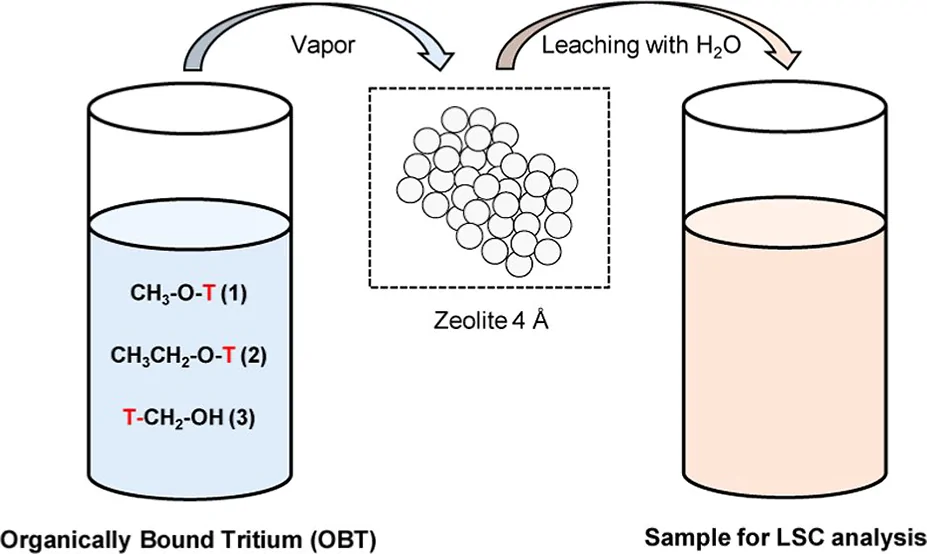

## Introduction

Tritium (T) is the established name of superheavy hydrogen (^3^H), a radioactive isotope with a half-life of 12.32 years that occurs both naturally and from anthropogenic sources.^1,2^ Since hydrogen is a biologically relevant element, tritium can become a dose-relevant nuclide under high-exposure scenarios, especially when present as organically bound tritium (OBT) as compared to tritiated water (HTO), which has a shorter biological half-life. Nonetheless, even releases of HTO, e.g., from the storage tanks of the Fukushima Daiichi nuclear power plant, are fiercely debated.^3^ Since tritium emits low-energy β^–^ particles with a mean energy of 5.7 keV^4^ and decays without any concurrent γ-photon emission, tritium monitoring is challenging^5^ and requires the transfer of the sample “into” the detector, which is typically achieved by sampling of material followed by liquid scintillation counting (LSC). This laborious procedure explains why tritium is an understudied radionuclide, even after major nuclear accidents such as Chornobyl^6,7^ or Fukushima.^8,9^ Especially in occupational exposure monitoring, a distinction between HTO and more hazardous OBT is necessary for risk assessment, thus prompting this study.

The synthesis of OBT compounds requires deep insight into the reaction mechanism as well as the choice of an apt synthesis method in order to achieve tritiation at the intended location of the molecule.^10,11^ Historically, tritium–protium exchange reactions were used for the synthesis of OBT compounds.^12,13^ There exists a plethora of research dealing with tritium exchange reactions, including reactions on various surfaces,^14^ such as catalysts^15^ for various applications.^16^ Tritium exchange reactions, however, can also result in an (unwanted) loss of tritium from a labeled compound, which could gradually reduce the specific activity of tritium from the OBT compound. Most of the previous research had focused on tritiated water.^14,17^ Here, as a blank experiment, we investigated the practical relevance of any tritium exchange reactions of tritiated low-molecular-weight alcohols on hydrated glass surfaces.

Tritium labeling plays a vital role in modern radiochemistry as well as in biochemical and pharmaceutical research.^10,11,18–20^ Such compounds have also been useful for other industrial applications. For example, low-molecular-weight alcohols tritiated at the carbon atom, such as CH_2_TOH, have been proposed as noncharged radiotracers in interwell studies.^21^ Due to their similarity to water and low tritium exchange rate, they are applied to monitor fluid communication.^22^ By contrast, alcohols tritiated at the oxygen atom are not necessarily equally suitable, particularly because their labeling position may be more prone to hydrogen exchange reactions. The different binding partners of the tritium atom within the molecule, here either carbon (C–T) or oxygen (O–T), play a vital role with respect to stability, as these bonds exhibit substantially different acidities^23^ and thereby different affinities toward tritium exchange.^24–26^ All protic organic molecules undergo an equilibrium reaction of tritium exchange under ambient conditions with HTO and other protic molecules.^24,26–28^ Historically, oxygen-substituted tritium compounds have seen few applications but are nowadays more important regarding waste management.^29^ Tritiated aliphatic alcohols with low molecular weight represent some of the most prominent waste forms in biomedical research involving tritium.^30^ Alcohols tritiated at the oxygen atom also pose a potential biological hazard since the tritium can be easily incorporated into the body due to rapid isotopic exchange with H_2_O and intake of the formed HTO.^29^

Both tritium decontamination^31–33^ and monitoring are equally important in the nuclear industry. Previous studies aiming at improved occupational exposure monitoring of tritium in high-tritium environments have exemplified the high suitability of zeolites for the monitoring of HTO vapor in air;^34,35^ however, the suitability for the monitoring of gaseous OBT has not been studied as deeply. Zeolites have been widely used as solid-phase sorbents for the removal of OBT compounds from liquid waste oils. This results in a transfer of activity to the sorbent and hence enables decontamination of the liquid phase.^36–38^ Detritiation of the gaseous phase, however, generally requires the oxidation of these OBT species to HTO vapor that can then be sorbed onto sorbents.^39,40^ Nevertheless, literature shows that zeolites are capable of a substantial uptake of low-molecular-weight organic molecules such as alcohols.^41–44^ Many volatile organic compounds (e.g., low-molecular-weight aliphatic alcohols) have a similarly small molecular size like H_2_O, hence motivating this study of the direct sorption (without prior oxidation) of used alcohols onto zeolites 4 Å. Most studies of the interaction of alcohols with zeolites have focused on the drying of alcohols.^45,46^ Mobasser et al. studied the uptake of volatile organic compounds by zeolite for air purification.^47^ To the best of our knowledge, however, no studies have been conducted investigating the uptake of tritiated low-molecular-weight alcohols by (small-pore) zeolites.

Here, we present a straightforward synthesis of low-molecular-weight tritiated molecules by transferring the activity of readily available aqueous solutions containing HTO into a simple organic molecule or by using other simple synthetic approaches that would yield a low specific activity of such molecules. In the first case, synthesis via hydrolysis of alkoxides yielded methan-1-ol-*O*-tritium (CH_3_OT, **1**) and ethan-1-ol-*O*-tritium (CH_3_CH_2_OT, **2**), whereas in the second case, the hydrolysis of a tritiated methyl nosylate yielded [1-^3^H]­methan-1-ol (CH_2_TOH, **3**). The purpose of this synthesis was to obtain activity concentrations that were comparable with previous environmental studies^21^ rather than to aim at a high degree of tritiation. The objective of this study was the use of these tritium-labeled alcohols to simulate the indoor atmospheric pollution of a nuclear facility and to test the response of zeolite-based water vapor samplers^34^ to low-molecular-weight OBTs.

Lastly, we tested any observable exchange of tritium atoms from our organic molecules by interaction with glass surfaces in order to ensure that no undesirable exchange of tritium atoms with surface-bound H_2_O molecules on glass surfaces would ultimately reduce the activity recovery.

## Experimental Section

### Synthesis of Tritiated Alcohols

Reagents, instruments, and suppliers are listed in the Supporting Information.

O-Tritiated alcohols (ROT) CH_3_OT (**1**) and CH_3_CH_2_OT (**2**) were synthesized by reaction of the respective alkoxides with a diluted aqueous HTO solution. C-Tritiated CH_2_TOH (**3**) was prepared by hydrolysis of [^3^H]­methyl-4-nitrobenzenesulfonate. A detailed protocol of the synthesis is given in the Supporting Information. Characterization was performed by using nuclear magnet resonance (NMR) spectroscopy on an inactive aliquot of **1** and **2** and radio-high performance liquid chromatography (radio-HPLC) on **3**, respectively (see Supporting Information). Activities were determined by using LSC.

### Tritium Exchange on Glass Surfaces

In order to test for any unwanted losses of activity, a blank experiment was set up and designed to simulate losses of T in the course of the synthesis by tritium exchange. In particular, it was investigated whether tritium exchange on hydrated glass surfaces would observably reduce the activity recovery in the course of the synthesis. For this experiment, 8 mL screw-neck glass vials were used. To ensure uniformity and reproducibility, the glass surface was etched with 2 mL of 65% nitric acid. The vials were sealed and kept in the dark for 3 h, which corresponded to the optimal etching time.^48^ This procedure ensured a clean glass surface, oxidized organic contaminants, decreased surface ions, protonated the surface, and formed Si–OH bonds.^48,49^ After thorough rinsing with distilled water and air-drying at room temperature, the vials were placed in a brand-new switched-off drying oven to ensure a closed environment. A 1 L glass bowl, filled with distilled water, was placed on the upper shelf of the said oven to create a saturated water vapor atmosphere. The vials were left undisturbed for 24 h to maximize the adsorption of water molecules on the glass surface.

Ten vials were placed in two rows of five each (Figure S1). Then, 2 mL of **1**, **2**, or **3** were pipetted into the first vial. After a contact time of 1 min, an aliquot (50 μL) was taken for LSC. After an additional 30 s, the remaining solution was pipetted into the next vial. This procedure was continued until the last of the ten vials was filled and an aliquot was taken. Afterward, the activity concentration of each vial was calculated and related to that of the first vial to assess whether a systematic decline would become observable. Finally, the alcohol was left in the last vial for 72 h to check for any long-term effects. The final activity was compared to the measured activity obtained after 1 min of exposure.

### Uptake of Small Tritiated Alcohols by Zeolite **4** Å

For the sorption experiments, zeolite 4 Å (2–5 mm, 30 g) was weighed in an 80 mL sealable polyethylene beaker. An 8 mL glass screw-neck vial was placed in the center of the zeolite bed. Subsequently, tritiated alcohol (**1**, **2**, or **3**, respectively, 2 mL each) was weighed into the vial. The experimental setup is shown in Figure S2; tabulated information is compiled in Table S4. The beaker was closed and kept at room temperature (22 °C) or at 60 °C in a drying oven for 24 h. The temperature of 60 °C was chosen to increase the fraction of alcohol molecules in the gas phase without gravely inhibiting their sorption.

After the 24 h exposure period, the screw-neck vial containing the alcohol was removed, and the zeolite was transferred to another sealable beaker. This ensured that only sorption from the gas phase would be accounted for and condensation would not interfere. The residual alcohol in each glass vial was weighed, and the amount evaporated was determined. The zeolites were then leached with triple distilled water (35 mL) for 24 h at room temperature. These conditions (including the mass and species of zeolite) were selected based on a systematic method optimization for the leaching of zeolites loaded with tritiated compounds, as conducted by Feng et al.^34^ An aliquot (10 mL) was taken with a syringe and filtered through a PDVF syringe filter (0.22 μm, ROTILABO). The activity of each aliquot was measured via LSC. As blank, fresh zeolite (30 g) was leached with triple distilled water (35 mL) under identical conditions. The activity of the entire leachate could be calculated and referenced to the activity of the evaporated alcohol. Further information on calculations and error propagation is provided in the Supporting Information. Due to the higher importance of **3** as a nonexchangeable radiotracer, the experiment was conducted three times for **3**, both at 22 °C and at 60 °C, for statistical reasons, whereas experiments for **1** and **2** were conducted once at each temperature.

## Results and Discussion

### Synthesis of Tritiated Alcohols

The synthesis of **1** yielded 6.92 g with an activity concentration of 214 ± 5 Bq mL^–1^ (corresponding to an activity yield of 31.8 ± 1.1%). For **2**, an activity concentration of 127.3 ± 1.7 Bq mL^–1^ and a mass of 9.93 g (activity yield 37.3 ± 1.3%) was achieved. The difference in yield between **1** and **2** is somewhat unexpected as the reactivity of sodium ethoxide toward hydrolysis is lower than that of sodium methoxide.^50^ This discrepancy is possibly due to experimental factors, such as longer distillation times or higher temperatures, leading to more complete hydrolysis of the sodium ethoxide and distillation of **2** rather than a chemical reason.

For the synthesis using [^3^H]­methyl-4-nitrobenzenesulfonate, a yield of 6.67 g of **3**, with an activity concentration of 1430 ± 40 Bq mL^–1^ (activity yield 7.2 ± 0.3%), was achieved. This low value can be attributed to the limited rate of neutral aqueous solvolysis of [^3^H]­methyl-4-nitrobenzenesulfonate under ambient conditions. Furthermore, the addition of the methanol leads to competition between the hydrolysis reaction and solvolysis by methanol. The latter is reported to proceed faster than the former, limiting the formation of **3**.^51^ This limitation can be mitigated by increasing the time between hydrolysis and methanol addition and/or increasing the temperature before the methanol addition.^51^ However, the low yield was not an issue because the product would not be carrier-free anyway and its activity concentration was already orders of magnitude higher than that of the previously synthesized ROT. Moreover, the byproduct of this reaction, [^3^H]­dimethyl ether, is highly volatile and thus was not expected to persist after the distillation step. The final activity concentrations (570 ± 15 Bq mL^–1^ for **3**, after dilution) were in a similar range to previous studies that suggested the use of HTO and tritiated alcohol for environmental applications (interwell studies).^21^ Therefore, this range was targeted for the present study.

### Characterization of Synthesized Alcohols

The head temperatures observed during distillation were 65 and 78 °C at atmospheric pressure for **1** and **2**, respectively. Literature values for the boiling points are 64.70 °C for methanol and 78.29 °C for ethanol.^52^ The chemical shifts in the ^1^H and ^13^C NMR spectra of synthesized inactive batches of methanol and ethanol (Figure S3–S6) show good agreement with the corresponding literature values.^53^ No noticeable impurities were present.

Following solvolysis of [^3^H]­methyl-4-nitrobenzenesulfonate, the radioactive product **3** was isolated by fractional distillation. The radioactivity was predominantly recovered in the fraction distilling at a head temperature of 65 °C at atmospheric pressure, which, again, is consistent with methanol’s boiling point. Furthermore, the radio-chromatogram of **3** shows a single peak at around 3.3 min under the applied conditions (Figure S7). No significant late-eluting radioactive species was present in the region where any residual starting material would be expected (Figure S8). This indicates that it was successfully removed during fractional distillation. Given the labeling at the methyl carbon atom and the hydrolysis workup, the side product 4-nitrobenzesulfonic acid is expected to be unlabeled and would, like residual toluene, only appear in the UV–vis trace, where no peak is visible. The other side product [^3^H]­dimethyl ether is too volatile to be recorded using radio-HPLC. These observations are therefore consistent with **3** as the main product with no significant radioactive or UV–vis-absorbing impurities detected.

The different synthesis routes were therefore suitable to receive the three target alcohols in sufficient purity as well as sufficient activity for the subsequent sorption experiments.

### Tritium Exchange on a Glass Surface

Comparing the activity concentration of the tritiated alcohols **1**, **2**, and **3** in each vial revealed no significant systematic decline as displayed in Figure 1. The deviation in the activity concentration relative to the first vial was smaller than the expanded uncertainty (*k* = 2) for all vials. This was consistent for **1**, **2**, and **3**. The activity of the alcohol in the last vial after 72 h was not significantly lower than that after 60 s contact time. Accordingly, under the given experimental conditions, no measurable tritium exchange on the glass surface for any of the three tritiated alcohols was observed, and 60 s contact time was sufficient. The results are also tabulated in Tables S1–S3 for **1**, **2**, and **3**, respectively. This observation is in accordance with the limited number of exchangeable protons on the glass surface in proportion to the large excess of alcohol molecules in the liquid phase. The existing literature shows that under ambient conditions, adsorption of water on glass is limited to a few molecular layers,^54^ corresponding to only a small number of molecules per unit area of glass. Moreover, even at maximum hydroxylation of the amorphous silica surface, the maximum density of silanol groups is reported to be 4.6–4.9 OH groups per nm^2^.^55^ The surface area of glass exposed to the tritiated alcohol was on the order of cm^2^ in this experiment. Therefore, the number of exchangeable protons originating from adsorbed water and surface silanol groups is negligible when exposed to an amount of alcohol large enough, as shown by the results. This would result in the equilibrium of the exchange reaction being overwhelmingly shifted toward the tritiated alcohol rather than tritiated water or tritiated silanol groups.

**Figure 1:**
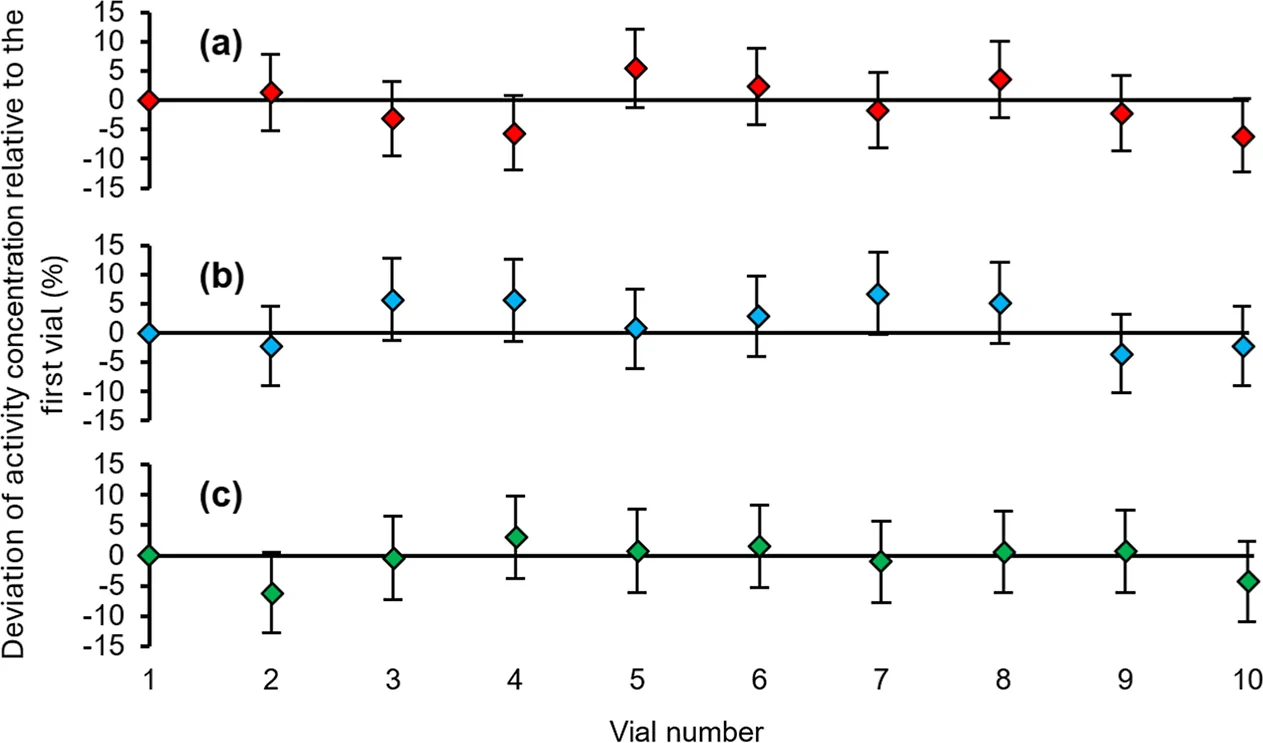
Results of the tritium exchange experiment on glass surfaces for (a) CH_3_OT (**1**), (b) CH_3_CH_2_OT (**2**), and (c) CH_2_TOH (**3**). The deviation of activity concentration in each vial relative to the activity concentration in the first vial in % is presented. The error bars indicate the expanded measurement uncertainty from LSC counting statistics and pipetting (*k* = 2).

If tritium exchange occurred on the glass surface at all, its extent would be too small to be resolved within the uncertainty of the applied LSC method and therefore does not affect the subsequent sorption experiments or the activity recovery during synthesis.

### Sorption of Alcohols onto Zeolite 4 Å

The sorption of the synthesized tritiated methanol and ethanol on zeolite 4 Å was examined at both room temperature (22 °C) and 60 °C. Figure 2a represents the recovered activity in the leachate in % of the activity of the evaporated alcohol in the closed beaker. Although there are slight deviations between the recovered activities of **1**, **2**, and **3** at the respective temperatures, the high-temperature experiment (60 °C) appears to consistently recover lower activities than the low-temperature experiments (20 °C). The marginal differences between the tritiated methanol species **1** and **3** are within uncertainty bars (*k* = 2) at their respective temperatures, but so is **2** as well. At lower temperatures, nearly all of the evaporated alcohol is effectively sorbed to the zeolite and thereby recovered in the leachate for LSC measurement. This proves the applicability of zeolite-based air monitoring samplers for low-molecular-weight alcohols at lower OBT concentrations in the air. At higher temperatures, the efficiency of the zeolite used in air samplers declines. This is likely the result of a combination of various phenomena: (1) reduced sorption to the zeolite (an exothermic reaction) and increasing desorption at higher temperature due to Le Chatelier’s principle; (2) partial, local saturation of the zeolite surface in our sorption experiment (Figure S2). Figure 2b shows the calculated mass of the sorbed alcohol per mass of zeolite present in the beaker. At higher temperatures (60 °C), a greater (absolute) amount of alcohol vapor is sorbed than at room temperature (22 °C). This is a result of the increased evaporation, causing a greater degree of saturation of the atmosphere inside the experimental beaker at 60 °C. Different volatilities explain certain differences in zeolite loading in methanol vs ethanol. The reasons for the different amounts of sorbed methanol (**1** vs **3**) per g of zeolite at 22 °C are not readily apparent and are likely linked to experimental factors, for example, slight deviations in the temperature profile, different volumes of the beaker, air moisture, etc. Please note that the experiment was conducted only once for **1** at both temperatures but three times for **3**. It is noteworthy that during the room temperature experiment, 0.780 ± 0.019 mL (*n* = 1; *k* = 1) of **1** had evaporated, whereas only 0.54 ± 0.10 mL (*n* = 3; *k* = 1) evaporated for **3**. Since the sorption of methanol by zeolite is almost quantitative at room temperature (as shown in Figure 2a), the staggering difference for the low-temperature profile of **1** and **3** in Figure 2b is already covered only by the different amount of evaporated methanol. The great variability of the amount of evaporated methanol is reflected by the relatively large standard deviation of 0.54 ± 0.10 mL.

**Figure 2:**
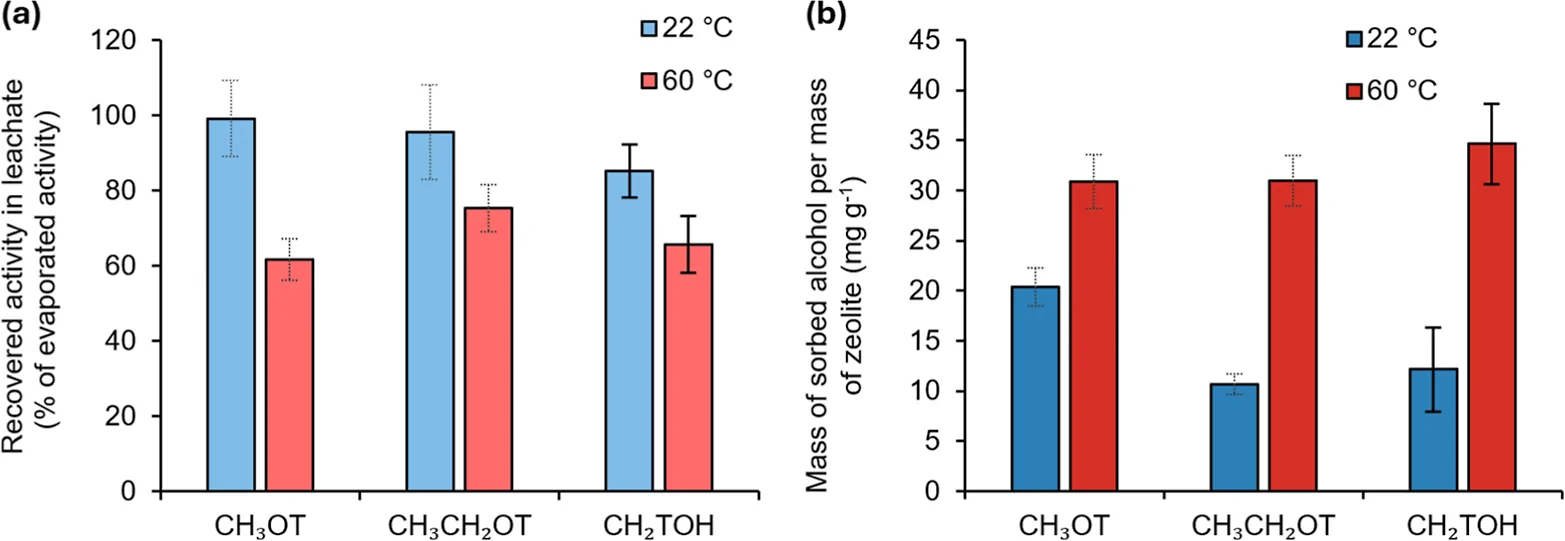
Results of the sorption experiments of CH_3_OT (**1**), CH_3_CH_2_OT (**2**), and CH_2_TOH (**3**) after exposure of zeolite 4 Å to tritiated alcohol vapor for 24 h at 22 °C (blue) and at 60 °C (red) followed by water leaching for 24 h at 22 °C. (a) Recovered activity in the leachate, normalized to the activity of the evaporated alcohol (*A*_leachate_/*A*_evaporated_ × 100%). Single measurements are shown for CH_3_OT (**1**) and CH_3_CH_2_OT (**2**); gray, dotted uncertainty bars represent propagated measurement uncertainty from LSC counting statistics, pipetting, and weighing (*k* = 2). For CH_2_TOH (**3**), mean values (*n* = 3) are presented; black, solid uncertainty bars represent standard deviation (*k* = 2). (b) Mass of sorbed alcohol per mass of zeolite (mg g^–1^) propagated from data in (a) (*m*_evaporated_/*m*_zeolite_ × *A*_leachate_/*A*_evaporated_). Error bars indicate propagated uncertainty (*k* = 2). Gray dotted lines correspond to *n* = 1 and black solid lines to *n* = 3.

The decrease in the relative recovery of the activity in the leachate with increased temperature, at first glance, might suggest that the evaporated alcohol has exceeded the effective capacity of the zeolite under the chosen conditions. However, literature reports significantly higher capacities of zeolite 4 Å for methanol and ethanol. For ethanol, Chaibi et al. reported an ethanol uptake of 175.6 mg g^–1^ at 20 °C and 169.6 mg g^–1^ at 45 °C after 4 h of adsorption determined by thermogravimetric analysis.^41^ Guo et al. simulated equilibrium loadings of 188.5 mg g^–1^ at 25 °C and 189.1 mg g^–1^ at 50 °C for pure ethanol.^42^ The observed sorption values in this study (10.7 ± 0.5 mg g^–1^ and 31.0 ± 1.3 mg g^–1^ for **2** at 22 and 60 °C, respectively) are thus considerably lower than what has been described in literature. A similar conclusion applies to tritiated methanol. Azhagapillai et al. suggested equilibrium uptake of 157 mg g^–1^ methanol at 33 °C and 247 mbar vapor pressure.^43^ The observed zeolite loadings in the present study are 20.3 ± 1.0 mg g^–1^ (at 22 °C) and 30.9 ± 1.4 mg g^–1^ (at 60 °C) for **1** as well as 12.1 ± 2.1 mg g^–1^ (at 22 °C) and 34.7 ± 2.0 mg g^–1^ (at 60 °C) for **3**, respectively.

Due to the application-focused approach in the experimental evaluation of the air sampling method, direct comparison of the zeolite loadings with reported literature values is challenging, as sorption depends (among other parameters such as zeolite condition and capacity) on both the partial pressure of the alcohol and temperature. These parameters were not controlled independently during the experiment because they neither would nor could be independently regulated or controlled under practical air sampling conditions. For the experiment at room temperature, the situation can be explained by the limited amount of vapor formed yet by a nearly quantitative sorption of tritiated alcohols (as indicated by a nearly quantitative recovery of activity). At 60 °C, the situation is reversed: despite increased partial vapor pressure, sorption was incomplete, although at lower loadings than suggested by literature. A plausible explanation for the limited activity recovery at 60 °C is likely to be found in the experimental setup. The beaker was filled with zeolite beads forming a layered arrangement. The sorption may predominantly happen in the uppermost layer of zeolite beads, while the lower layers remain underexposed to the vapor. According to this theory, the beads in the top region may have already reached sorption equilibrium during the high-temperature experiment, whereas the bottom beads exhibit lesser uptake. This could impair further sorption through the layers of beads, which negatively impacts the relative recovery of activity in the leachate. Another contributing factor might be the uncontrolled amount of water present due to air humidity and residual moisture of the alcohol, which would reduce sorption capabilities for ethanol as water–ethanol mixtures exhibit lower equilibrium loadings.^42^ Any disparity in sorption due to size and vapor pressure differences as a result of tritium labeling (note that literature values are for nontritiated compounds) in our diluted system is unobservably small.

The results provide clear trends for the sorption of tritiated low-molecular-weight alcohols. However, with a limited amount of tritiated material available, a prioritization for the experiments was critical, causing a natural limitation of repetitions. The results, nonetheless, indicate the suitability of the zeolite 4 Å-based air monitors for the atmospheric monitoring of OBT alcohols. Certain, unavoidable environmental fluctuations represented boundaries in the experiments that were difficult to fully control. These boundaries, however, do not impact the overall qualitative applicability of the sampling method, which, for example, proved robust even against significant temperature changes.

## Conclusions

In this study, the applicability of zeolite 4 Å-based air monitors was successfully tested for their response toward tritiated low-molecular-weight alcohol vapor. Tritiated alcohols included C- and O-bound T methanol (**1** and **3**, respectively) and O-bound T ethanol (**2**). At room temperature (22 °C), alcohol sorption by the zeolite is nearly quantitative, as shown by a nearly complete recovery of the activity of the evaporated alcohols via a leaching technique. At higher temperature (60 °C), activity recovery decreases down to 62 ± 6% for **1**, 75 ± 8% for **2**, and 66 ± 8% for **3** (*k* = 2), respectively. At the same time, when increasing the temperature from 22 to 60 °C, the zeolite loading of sorbed alcohols increases (mass of sorbed alcohol per mass of zeolite) from 20.3 ± 2.0 mg g^–1^ to 30.9 ± 2.8 mg g^–1^ for **1**; from 10.7 ± 1.0 mg g^–1^ to 31.0 ± 2.6 mg g^–1^ for **2**; and from 12.1 ± 2.1 mg g^–1^ to 34.7 ± 2.0 mg g^–1^ for **3**. Please note that these results are highly dependent on the environmental and experimental boundary conditions. For quantitative monitoring of OBT compounds, a custom-made calibration of the zeolite, depending on factors such as ambient temperature, humidity, and volume of the monitored space, will become necessary. While the zeolite in our experimental setup was well suited for low-molecular-weight alcohol molecules, the efficiency of a monitoring setup will also depend on the chemical species of the OBT molecule in question. Blank experiments revealed no observable exchange of T upon interaction of the tritiated alcohols with hydrated glass surfaces, neither in exchangeable form (**1**, **2**) nor in non-exchangeable form (**3**).

## Supplementary Material



## Notes

Hydrolysis reaction of [^3^H]­methyl-4-nitrobenzenesulfonate (Scheme S1); experimental setup for ^3^H exchange experiments on glass surfaces (Figure S1); experimental setup of the sorption experiment (Figure S2); ^1^H NMR spectrum of methanol (Figure S3); ^13^C NMR spectrum of methanol (Figure S4); ^1^H NMR spectrum of ethanol (Figure S5); ^13^C NMR spectrum of ethanol (Figure S6); radio-HPLC chromatograms of synthesized CH_2_TOH (Figure S7); radio-HPLC chromatograms of [^3^H]­methyl-4-nitrobenzenesulfonate in toluene (Figure S8); results of the ^3^H exchange experiments on glass surfaces for (**1**), (**2**), and (**3**), respectively (Tables S1–S3); parameters for sorption experiments of tritiated alcohols on zeolite 4 Å (Table S4); quantitative results of sorption experiments of tritiated alcohol on zeolite 4 Å (Table S5); reagents, instruments, and their suppliers; synthesis and characterization protocols of the title compounds; characterization of the title compounds and starting materials; and calculations and error propagation (PDF)
