# Supplementary material for: Retention of Tritiated Alcohols by Zeolites Used in Indoor Air Sampling
Source: ACS Omega. 2026 Sep 24;11(39):60371–7. doi: 10.1021/acsomega.6c08364 (PMC13647871; doi:10.1021/acsomega.6c08364)
Supplement: Supplementary file 1 [file ao6c08364_si_001.pdf]

Supporting Information

Retention of Tritiated Alcohols by Zeolites Used in Indoor Air Sampling

Lukas Zellinger,[1] Dominik Boya,[1] Veronika Rosecker,[1] Bin Feng,[2] Georg Steinhauser[1,*]

[1]Institute of Applied Synthetic Chemistry & TRIGA Center Atominstitut, Technische Universität Wien, Vienna 1060, Austria

[2] Institute of Radiation Medicine, Fudan University, Shanghai 200437, China

[*] Corresponding author. georg.steinhauser@tuwien.ac.at

Reagents, Instruments, and Suppliers

Unless otherwise noted, all reagents were purchased from commercial suppliers and used without further purification. Reactions were carried out in air-dried glassware with magnetic stirring. The used chemicals were Uvasol® grade methanol (≥99.9 % purity) and Emsure® grade ethanol (≥99.9 % purity) both from Merck™ KGaA (Darmstadt, Germany); sodium metal (99.5 % purity) from Riedel de Haën™ AG (Seelze, Germany); anhydrous calcium chloride (granular, ≥93 % purity) from Sigma-Aldrich™ (St. Louis, MO, USA); ROTIPURAN® grade nitric acid (≥65 %, p.a.), ROTISOLV® HPLC grade toluene (≥99.9 % purity), ROTISOLV® HPLC gradient grade acetonitrile (MeCN) (≥99.9 % purity), ROTIPURAN® LCMS grade trifluoroacetic acid (TFA) (≥99.9 % purity), and zeolite 4 Å (2–5 mm) were all obtained from Carl Roth (Karlsruhe, Germany). For the drying of the products, powdered molecular sieve 3 Å from Merck™ KGaA (Darmstadt, Germany) was used after pre-activation (heating at 300 °C overnight and cooling in a desiccator over calcium chloride). The tritiated water (HTO) used in this study is a historic tritium sample that was obtained from the radionuclide storage of TU Wien and is decades old (16580 ± 40 Bq mL^{-1} at the time of the experiments, as determined by the measurement of a 50 µL aliquot). No further specifications beyond the activity are available. [^{3}H]Methyl-4-nitrobenzenesulfonate was obtained from RC Tritec (Teufen, Switzerland). 8 mL screw-neck glass vials were purchased from VWR International. For the radioanalytical quantification, the following equipment was used: Revvity™ Ultima Gold LLT liquid scintillation cocktail ("cocktail"), a Hidex™ 300 SL liquid scintillation counting (LSC) system, and 20 mL polyethylene LSC vials (mixture of sample and cocktail in a ratio of typically either 1:1 for the leachate measurements or 1:200 for all other samples). Samples were measured in ^{3}H pre-settings (window 5–30) and counted for 1 h after a tray delay of 2 h, in which the sample was left in the dark to minimize chemiluminescence and photoluminescence effects.[1] Each measurement was repeated twice and averaged. Samples were accompanied by a blank of triple distilled water. ^{1}H and ^{13}C NMR spectra were recorded at room temperature on a Bruker™ Advance UltraShield. Radio-HPLC analysis was performed on an UltiMate™ 3000 system (Thermo Fisher Scientific) equipped with an Eclipse Plus C18 (2.1 × 100 mm, 3.5 mikron) column.

Gradient elution from 10–95 % MeCN + TFA 0.1 %/water + TFA 0.1 % was applied over 20 min at a flow rate of 0.8 mL min^{-1}. Detection was performed with a Variable Wavelength Detector VWD-3400 at 254 nm and a FlowStar² LB 514 radioactivity detector (Berthold) using a solid scintillator cell YG 150-U60.

Synthesis of CH_3OT (1) and CH_3CH_2OT (2)

For the synthesis of **1** and **2,** the respective alcohols were deprotonated using sodium metal, forming the corresponding sodium alkoxides. The synthesis conducted was based on the synthesis of sodium alkoxides described by Chandran K. et al.[2] with some adaptions. In a three-neck-round-bottom flask equipped with a thermometer and Dimroth condenser with a desiccant cartridge filled with anhydrous calcium chloride and a bubbler, methanol (40 mL, 1.2 mol) or ethanol (40 mL, 0.87 mol) was heated to reflux. Thin pieces of sodium were added, each after the previous piece had completely dissolved. Sodium was added until saturation was reached. In total, 5.3 g (0.23 mol) of sodium were added for the synthesis of sodium methoxide and 3.6 g (0.16 mol) for sodium ethoxide. An additional aliquot of the alcohol (5 mL) was added to dissolve any remaining sodium. Excess alcohol was removed via rotary evaporation, although complete dryness may not have been achieved. In any case, the aim was not to yield a carrier-free tritiated alcohol, as the overwhelming matrix in the HTO-containing liquid was H_2O. Inactive alcohols would also form during hydrolysis with pure HTO as well. Therefore, no isotopically pure compounds of **1** or **2** were to be expected.

An aliquot of each alkoxide (10 wt%) was taken for later characterization. The remaining alkoxides were hydrolyzed with HTO (16580 ± 40 Bq mL^{-1}), 3.75 mL (0.209 mol) for the synthesis of **1** and 2.55 mL (0.142 mol) to obtain the desired tritiated alcohols **1** and **2**. Due to the exothermic nature of the hydrolysis, the sodium alkoxide (15.0 g, 0.208 mol methoxide; 18.4 g, 0.142 mol ethoxide) was placed in a flask of a distillation apparatus. HTO was added and evaporation of the formed tritiated alcohol started immediately.

The resulting alcohol was distilled with a 160 mm distillation unit right after HTO was added. Due to the exothermic nature of this reaction, the released energy was initially

enough to evaporate the alcohol once it had formed. After some time, additional heating was necessary to ensure complete distillation (65 °C for methanol and 78 °C for ethanol). The resulting sodium hydroxide and unreacted sodium alkoxide remained in the solid residue, whereas the tritiated alcohol was collected as the distillate. The alcohols **1** and **2** were dried over pre-activated powdered molecular sieve 3 Å overnight on a shaking table. The alcohols **1** and **2** were distilled again and yielded partially tritiated methanol (7.54 g) and partially tritiated ethanol (11.2 g) as the final product.

Presenting alcohol in large excess yielded the best results, as the limiting factor for the reaction was the solubility of the alkoxide in the alcohol. When excessive amounts of sodium were added, the respective alkoxide would precipitate on the surface of unreacted sodium, thereby decreasing reactivity as observed through reduced bubble formation in the bubbler. The formation of sodium hydroxide due to contact of sodium with moisture[2] was not considered problematic, as it would form in later steps of the synthesis of ROT anyway.

Synthesis of CH_2TOH (3)

Synthesis of **3** was started from commercially available [^{3}H]methyl-4-nitrobenzenesulfonate used previously as radio-HPLC references. Hydrolysis was performed by addition of triple distilled water (800 µL) to [^{3}H]methyl-4-nitrobenzenesulfonate dried in HPLC-vials. A total of seven previously used references were used for the synthesis of **3**. After 5 h, the aqueous phases were combined and 0.51 mL of the aqueous solution were mixed with methanol (10 mL) as carrier. The aqueous solution had an activity concentration of 327 ± 8 kBq mL^{-1} (50 µL were measured in 10 mL cocktail), giving a total yield of 1.80 ± 0.04 MBq after hydrolysis (Scheme S1). **3** was purified from the resulting byproducts, non-reacted [^{3}H]methyl-4-nitrobenzenesulfonate and water by fractional distillation. The distillate received was treated in the same manner as described above. **3** was diluted with methanol to adjust its activity concentration to be within the same order of magnitude as ROT.

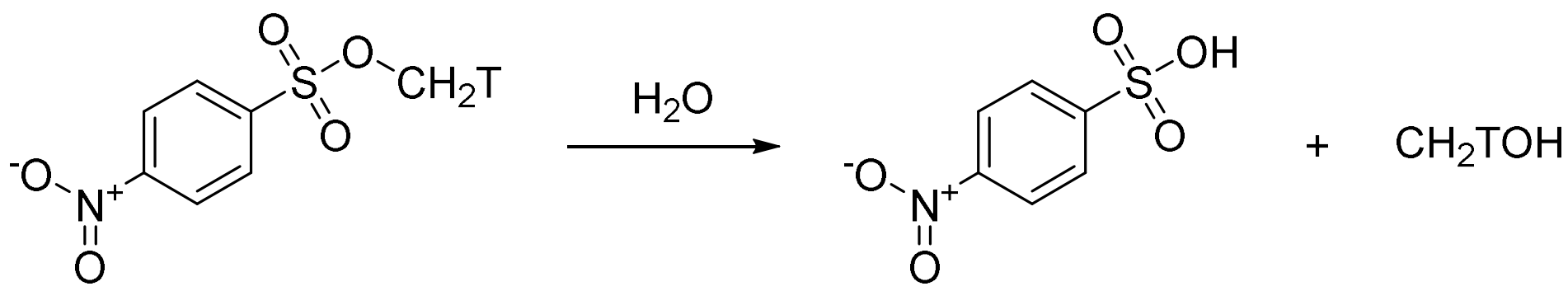

Scheme S1 Hydrolysis reaction of [^{3}H]methyl-4-nitrobenzenesulfonate

Characterization of tritiated alcohols

The synthesized tritiated alcohols were characterized using radio-HPLC on the same system as described in the SI using 10–90 % MeCN + TFA 0.1 %/water + TFA 0.1 % gradient elution over 14 min preceded by a 2 min pre-equilibration step at a flow rate of 0.8 mL min^{-1}. However, due to the low activity concentration of **1** and **2** no signal was obtained and only **3** (CH_2TOH) could be characterized this way.

Therefore, alternative NMR characterization was chosen, but since radioactive samples were not allowed, an indirect characterization was performed. An aliquot of the respective sodium alkoxide was hydrolyzed with non-tritiated triple distilled water instead of HTO and the subsequent work-up performed identical to tritiated samples. The received ROH (100 µL) was diluted with deuterated water (600 µL) and characterized via ^{1}H and ^{13}C NMR-spectroscopy. The chemical shifts were referenced to the residual H_2O signal.

Tritium exchange on glass surface

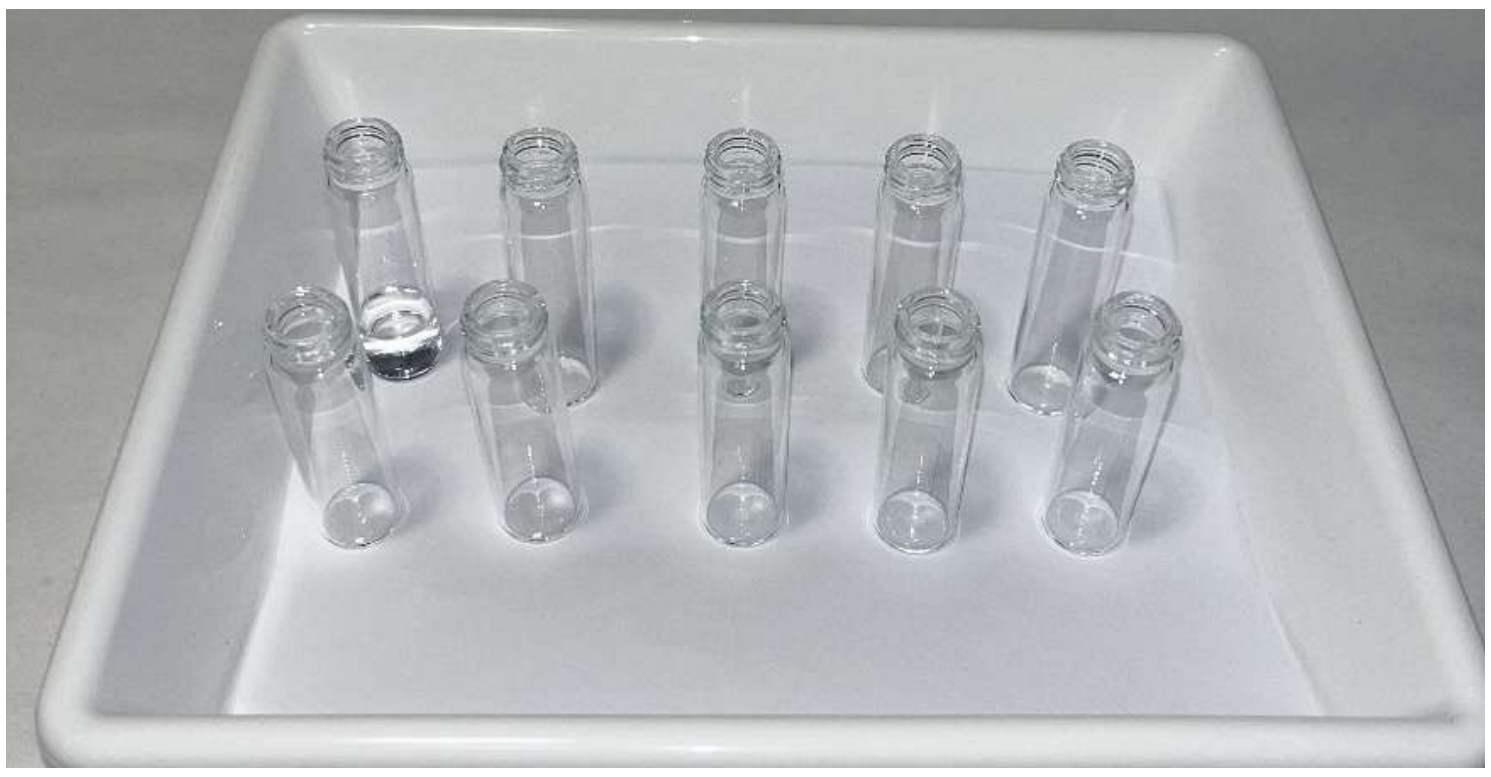

Fig. S1 Experimental setup for the tritium exchange experiment on a glass surface

Table S1 Results of the tritium exchange experiment on a glass surface for CH_3OT

	Activity concentration with propagated measurement uncertainty ($k = 1$)	Deviation of activity concentration relative to the first vial with expanded propagated measurement uncertainty ($k = 2$)
Vial 1	238 ± 6 Bq mL^{-1}	0 ± 0 %
Vial 2	242 ± 6 Bq mL^{-1}	1 ± 7 %
Vial 3	231 ± 6 Bq mL^{-1}	-3 ± 7 %
Vial 4	225 ± 6 Bq mL^{-1}	-6 ± 7 %
Vial 5	252 ± 6 Bq mL^{-1}	6 ± 7 %
Vial 6	244 ± 6 Bq mL^{-1}	2 ± 7 %
Vial 7	234 ± 6 Bq mL^{-1}	-2 ± 7 %
Vial 8	247 ± 6 Bq mL^{-1}	4 ± 7 %
Vial 9	233 ± 6 Bq mL^{-1}	-2 ± 7 %
Vial 10	224 ± 6 Bq mL^{-1}	-6 ± 7 %
Vial 10 after 72 h	233 ± 6 Bq mL^{-1}	-2 ± 7 %

Table S2 Results of the tritium exchange experiment on a glass surface for CH_3CH_2OT

	Activity concentration with propagated measurement uncertainty ($k = 1$)	Deviation of activity concentration relative to the first vial with expanded propagated measurement uncertainty ($k = 2$)
Vial 1	116 ± 3 Bq mL^{-1}	0 ± 0 %
Vial 2	113 ± 3 Bq mL^{-1}	-6 ± 7 %
Vial 3	122 ± 3 Bq mL^{-1}	0 ± 7 %
Vial 4	122 ± 3 Bq mL^{-1}	3 ± 7 %
Vial 5	116 ± 3 Bq mL^{-1}	1 ± 7 %
Vial 6	119 ± 3 Bq mL^{-1}	2 ± 7 %
Vial 7	123 ± 3 Bq mL^{-1}	-1 ± 7 %
Vial 8	121 ± 3 Bq mL^{-1}	1 ± 7 %
Vial 9	111 ± 3 Bq mL^{-1}	1 ± 7 %
Vial 10	113 ± 3 Bq mL^{-1}	-4 ± 7 %
Vial 10 after 72 h	116 ± 3 Bq mL^{-1}	-2 ± 7 %

Table S3 Results of the tritium exchange experiment on a glass surface for CH_2TOH

	Activity concentration with propagated measurement uncertainty ($k = 1$)	Deviation of activity concentration relative to the first vial with expanded propagated measurement uncertainty ($k = 2$)
Vial 1	1690 ± 40 Bq mL^{-1}	0 ± 0 %
Vial 2	1590 ± 40 Bq mL^{-1}	-2 ± 7 %
Vial 3	1680 ± 40 Bq mL^{-1}	6 ± 8 %
Vial 4	1730 ± 40 Bq mL^{-1}	6 ± 8 %
Vial 5	1700 ± 40 Bq mL^{-1}	1 ± 7 %
Vial 6	1710 ± 40 Bq mL^{-1}	3 ± 7 %
Vial 7	1670 ± 40 Bq mL^{-1}	7 ± 8 %
Vial 8	1700 ± 40 Bq mL^{-1}	5 ± 8 %
Vial 9	1700 ± 40 Bq mL^{-1}	-4 ± 7 %
Vial 10	1620 ± 40 Bq mL^{-1}	-2 ± 7 %
Vial 10 after 72 h	1650 ± 40 Bq mL^{-1}	9 ± 8 %

Sorption onto Zeolite 4 Å

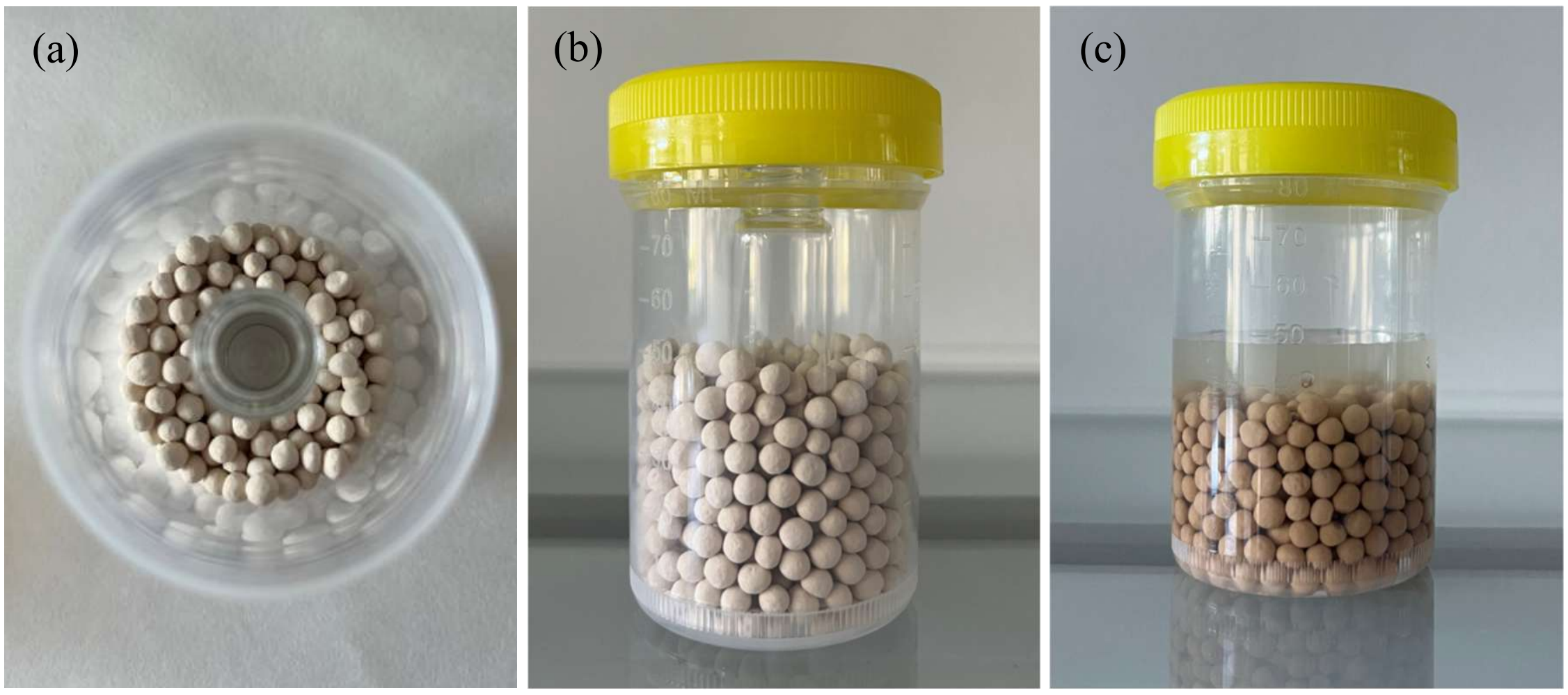

Fig. S2 Experimental setup of sorption experiment. (a) top view, open, before alcohol addition; (b) side-view, closed, after alcohol addition; (c) side-view, closed after leaching with triple distilled water

Table S4 Parameters for sorption experiments of tritiated alcohols on zeolite 4 Å (presented measurement uncertainty k = 1).

Alcohol added to vial	Temperature	Mass of added zeolite 4 Å	Volume of evaporated alcohol	Activity of evaporated alcohol
CH_3OT	22 °C	29.99730 ± 0.00009 g	0.780 ± 0.020 mL	167 ± 6 Bq
CH_3OT	60 °C	30.07524 ± 0.00009 g	1.911 ± 0.020 mL	409 ± 11 Bq
CH_3CH_2OT	22 °C	29.94347 ± 0.00009 g	0.423 ± 0.020 mL	50 ± 3 Bq
CH_3CH_2OT	60 °C	29.88550 ± 0.00009 g	1.557 ± 0.020 mL	185 ± 6 Bq
CH_2TOH replicate 1	22 °C	29.55000 ± 0.00009 g	0.638 ± 0.020 mL	182 ± 7 Bq
CH_2TOH replicate 2	22 °C	29.68460 ± 0.00009 g	0.524 ± 0.020 mL	149 ± 7 Bq
CH_2TOH replicate 3	22 °C	29.93940 ± 0.00009 g	0.448 ± 0.020 mL	128 ± 7 Bq
CH_2TOH replicate 1	60 °C	29.99260 ± 0.00009 g	2.000 ± 0.020 mL	570 ± 15 Bq
CH_2TOH replicate 2	60 °C	29.94500 ± 0.00009 g	2.000 ± 0.020 mL	570 ± 15 Bq
CH_2TOH replicate 3	60 °C	29.95830 ± 0.00009 g	2.000 ± 0.020 mL	570 ± 15 Bq

Table S5 Quantitative results of sorption experiments of tritiated alcohol on zeolite 4 Å (presented propagated measurement uncertainty $k = 1$).

Alcohol added to vial	Temperature	Activity of the entire leachate	Recovered activity in leachate relative to evaporated activity	Mass of sorbed alcohol per mass of zeolite
CH_3OT	22 °C	166 ± 7 Bq	99 ± 6 %	20.3 ± 1.0 mg g^{-1}
CH_3OT	60 °C	252 ± 10 Bq	62 ± 3 %	30.9 ± 1.4 mg g^{-1}
CH_3CH_2OT	22 °C	48.1 ± 2.0 Bq	96 ± 7 %	10.7 ± 0.5 mg g^{-1}
CH_3CH_2OT	60 °C	139 ± 5 Bq	75 ± 4 %	31.0 ± 1.3 mg g^{-1}
CH_2TOH replicate 1	22 °C	155 ± 9 Bq	85 ± 6 %	14.5 ± 0.9 mg g^{-1}
CH_2TOH replicate 2	22 °C	122 ± 7 Bq	82 ± 6 %	11.4 ± 0.7 mg g^{-1}
CH_2TOH replicate 3	22 °C	113 ± 7 Bq	89 ± 7 %	10.5 ± 0.7 mg g^{-1}
CH_2TOH 22°C Mean ± SD ($k = 1$)			85 ± 4 %	12.1 ± 2.1 mg g^{-1}
CH_2TOH replicate 1	60 °C	358 ± 16 Bq	63 ± 4 %	33.1 ± 1.7 mg g^{-1}
CH_2TOH replicate 2	60 °C	367 ± 16 Bq	64 ± 4 %	34.0 ± 1.7 mg g^{-1}
CH_2TOH replicate 3	60 °C	399 ± 19 Bq	70 ± 4 %	36.9 ± 2.0 mg g^{-1}
CH_2TOH 60°C Mean ± SD ($k = 1$)			66 ± 4 %	34.7 ± 2.0 mg g^{-1}

Characterization of starting material and products

The recorded NMR spectra of the synthesized methanol and ethanol are shown in Fig. S3–S6. The received codes for the synthesized methanol are: ^{1}H NMR (400 MHz, D_2O) δ = 3.33 (s, 3H); ^{13}C NMR (101 MHz, D_2O) δ = 48.77. For ethanol they are: ^{1}H NMR (400 MHz, D_2O) δ = 3.62 (q, J = 7.1 Hz, 2H), 1.15 (t, J = 7.1 Hz, 3H); ^{13}C NMR (101 MHz, D_2O) δ = 57.34, δ = 16.77. The letters s (singlet), q (quartet) and t (triplet) depict the multiplicities, J the coupling constants and δ the chemical shifts in ppm relative to the residual H_2O peak.

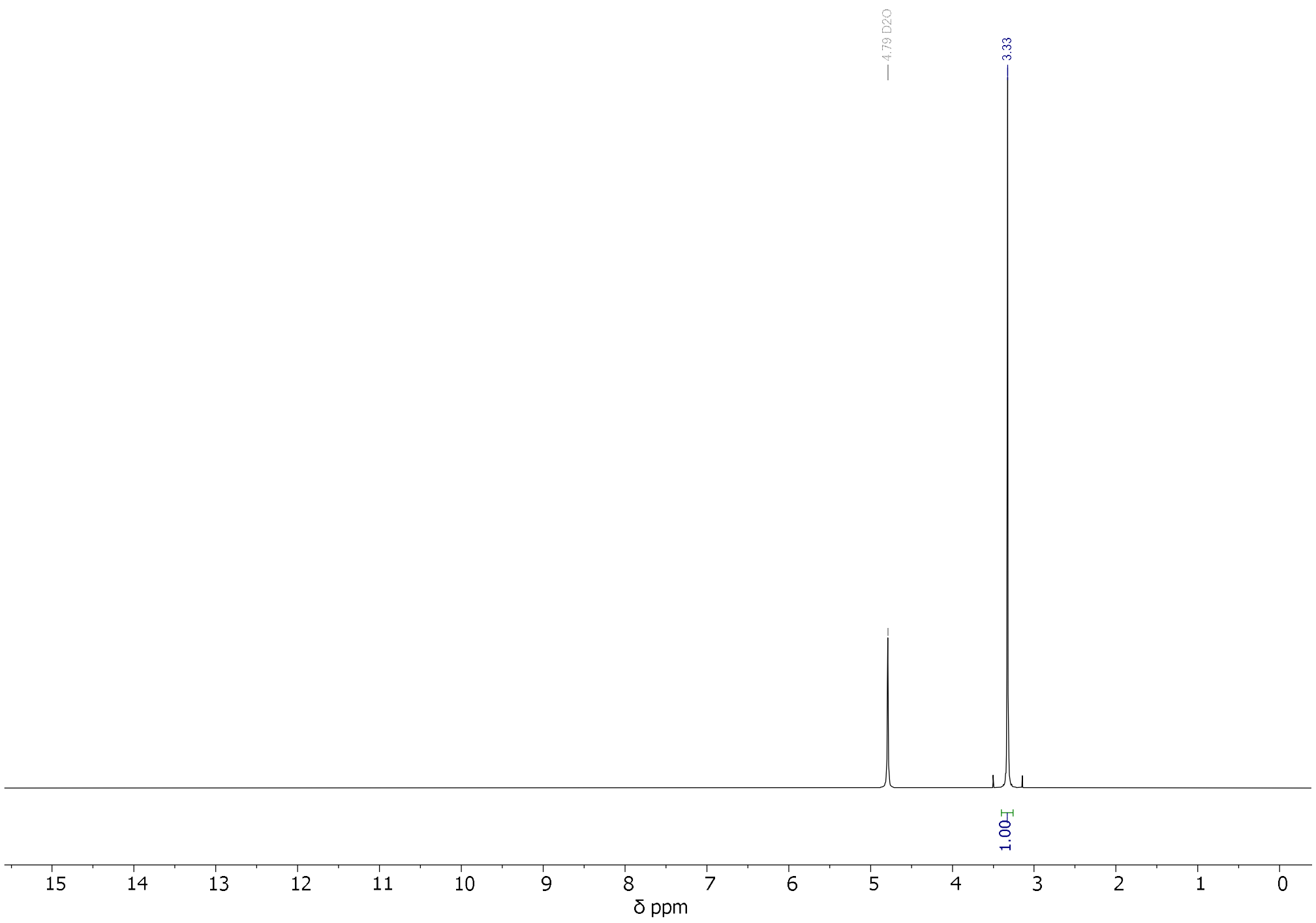

Fig. S3 ^{1}H NMR spectrum of synthesized methanol (400 MHz, D_2O)

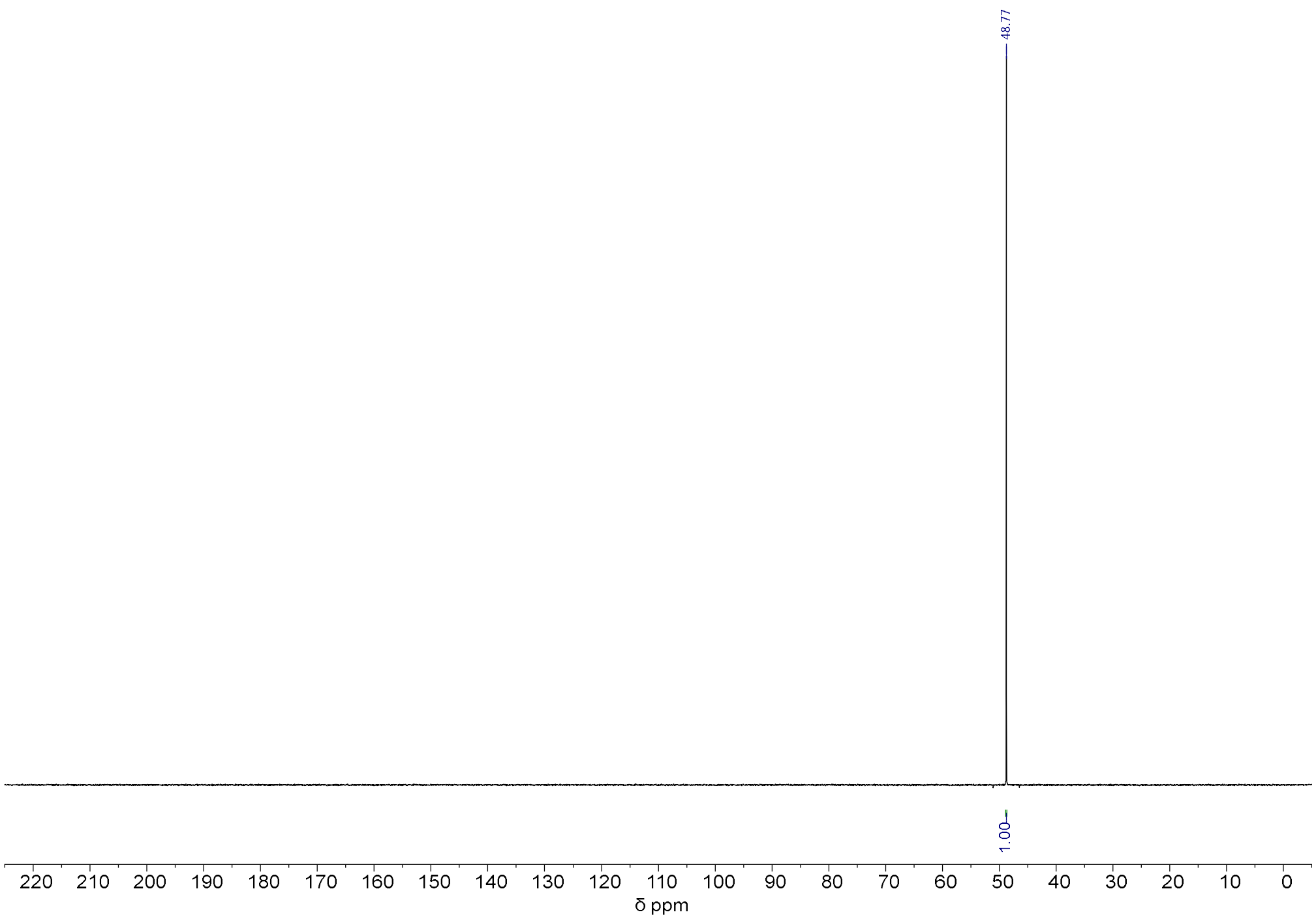

Fig. S4 ^{13}C NMR spectrum of synthesized methanol (101 MHz, D_2O)

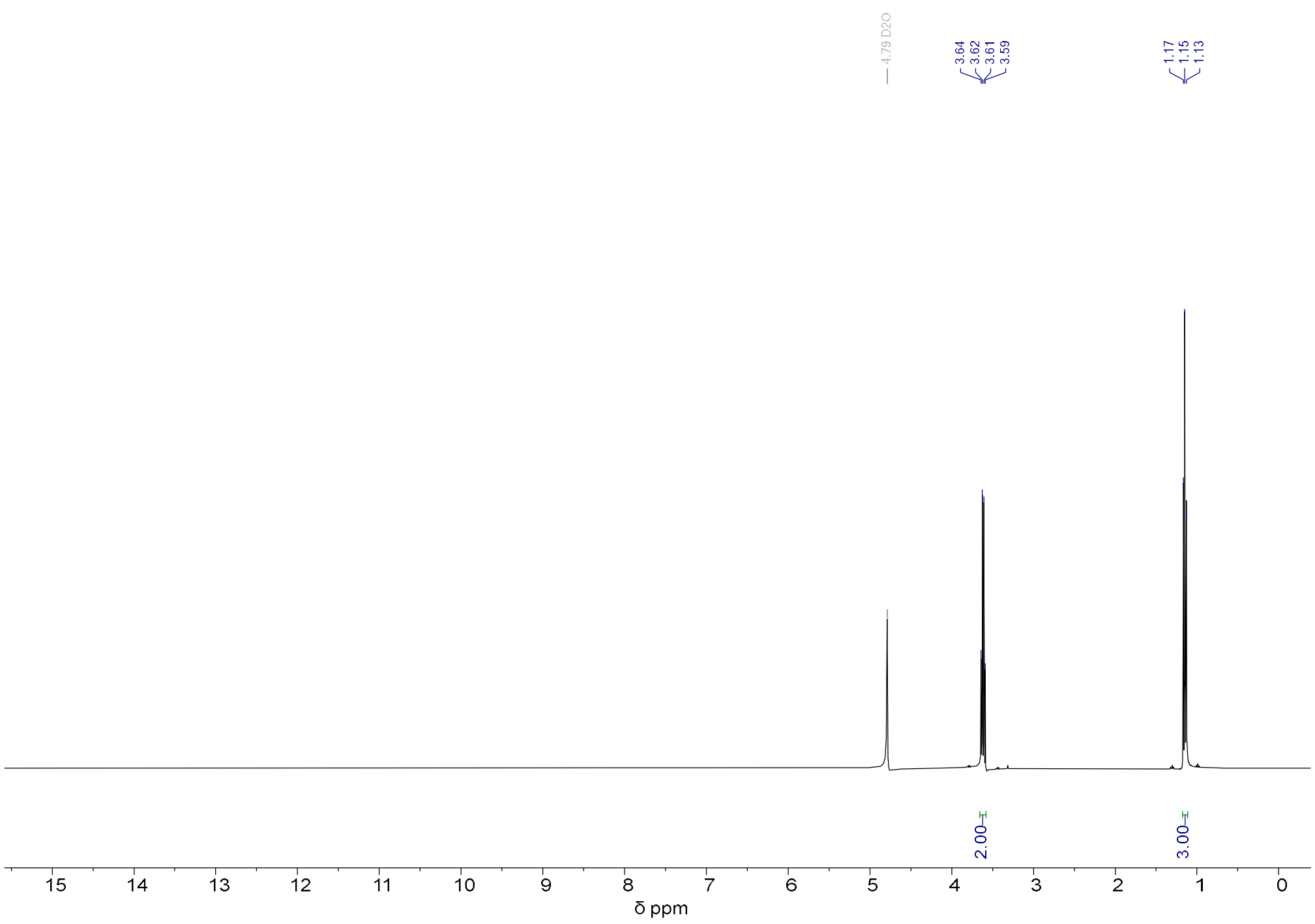

Fig. S5 ^{1}H NMR spectrum of synthesized ethanol (400 MHz, D_2O)

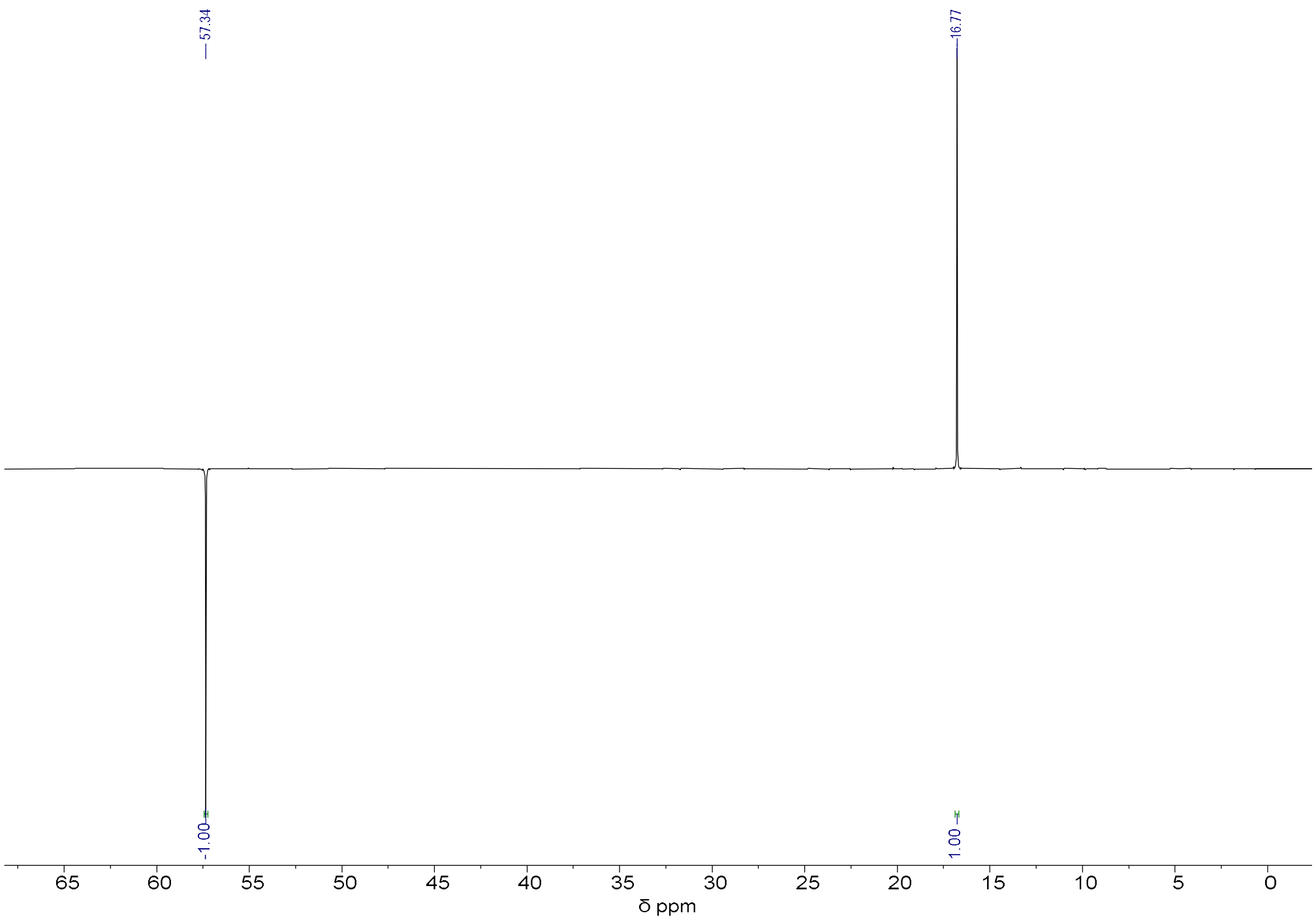

Fig. S6 ^{13}C NMR spectrum of synthesized ethanol (101 MHz, D_2O)

Fig. S7 shows the count rate and UV-vis trace of the radio-HPLC measurement of the synthesized CH_2TOH. Both count rate and UV-vis trace are presented.

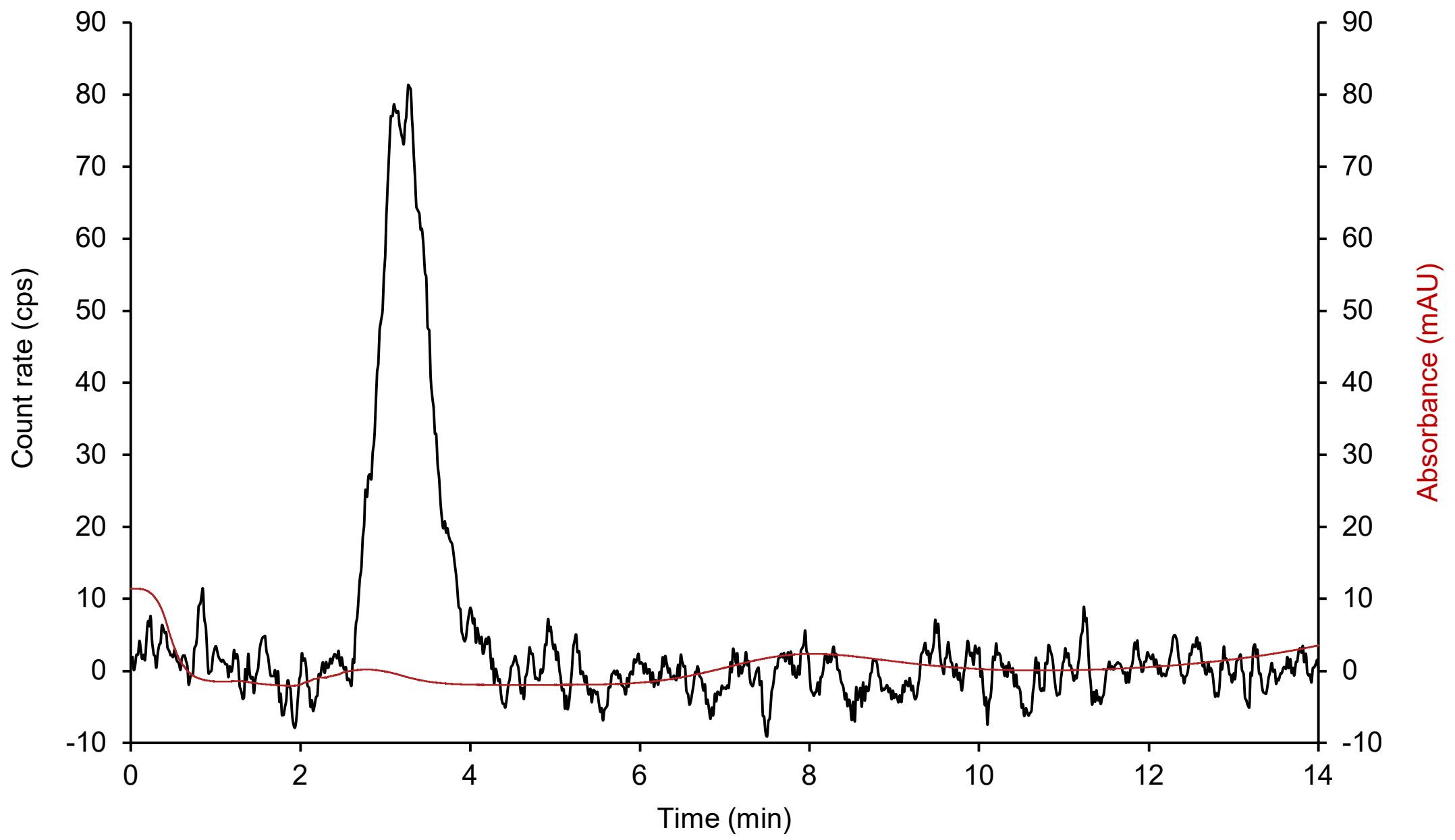

Fig. S7 Radio-HPLC chromatograms of synthesized CH_2TOH under identical chromatographic conditions. Radioactivity trace (left, black axis) and UV-vis trace (right, red axis). Both signals were baseline-corrected by subtraction of the average signal in a peak-free region.

The radio-HPLC analysis of the later hydrolyzed [^{3}H]methyl-4-nitrobenzenesulfonate yielded the chromatogram displayed in Fig. S8. Once again both count rate and UV-vis trace are presented. Radio-HPLC analysis of the precursor showed a single predominant peak in the radiotrace at 11.95 min, which correlates with a radiochemically pure starting material. The other peak in the chromatogram, which appears at 15.19 min in the UV-vis trace, originates from the solvent toluene. Under reversed-phase conditions, toluene exhibits a higher retention time than [^{3}H]methyl-4-nitrobenzenesulfonate, consistent with the higher polarity of the latter.

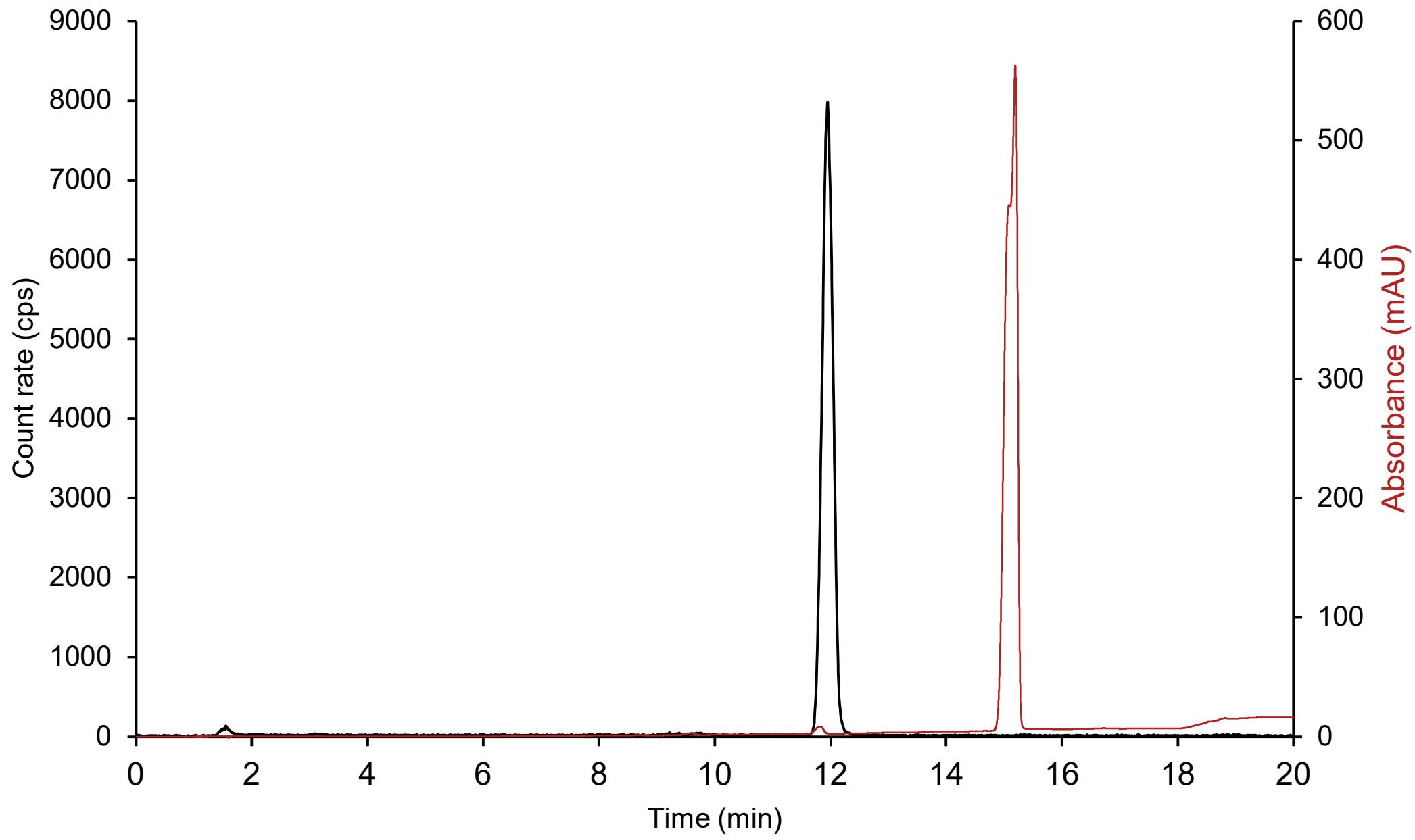

Fig. S8 Radio-HPLC chromatograms of [^{3}H]methyl-4-nitrobenzenesulfonate in toluene under identical chromatographic conditions. Radioactivity trace (left, black axis) and UV-vis trace (right, red axis).

Calculations and error propagation

The activity concentration a (Bq mL^{-1}) was calculated following Eq. S1 where C_s and C_b are the count rate of sample and blank, respectively (cpm) and V_s is the sample volume added to the vial (mL). The LSC was calibrated using ^3H reference solutions (IAEA) and quenching agent (CCl_4) to create a quenching curve as described by Feng et al.[3] The measuring efficiency is given by ε.

$$a = \frac{A}{V_\mathrm{t}} = \frac{C_\mathrm{s} - C_\mathrm{b}}{60\varepsilon\, V_\mathrm{s}} \tag{S1}$$

The activity A (Bq) of the sample was calculated by multiplying the activity concentration a with the total sample volume V_t (mL).

The uncertainty of the activity concentration $U(a)$ and activity $U(A)$ were calculated taking into account each contributing factor. For mass measurements and pipetted volumes uncertainties were taken from the manufacturer specifications of the respective balances and pipettes (Sartorius, VWR, Eppendorf). The uncertainty for the measuring efficiency was approximated using a 95 % confidence interval for the quenching curve. The statistical uncertainty of the count rates $U(C_i)$ are calculated assuming Poisson statistics following Eq. S2 where C_i is the count rate (cpm) and t_i the counting time (min).

$$U(C_i) = \sqrt{\frac{C_i}{t_i}} \tag{S2}$$

The radiochemical yield RCY (%) was calculated by Eq. S3 where A_p and A_0 represent the activity of the product and the reagent, respectively (Bq). A_p was decay-corrected to the reference time of the measurement of A_0, using the half-life of tritium ($T_{1/2} = 4497 \pm 4$ days)[4] and the elapsed time interval $t_{\mathrm{p},0}$ (d).

$$RCY = \frac{A_\mathrm{p}}{A_0} \exp\left(\frac{\ln 2}{T_{1/2}} t_{\mathrm{p},0}\right) \times 100\ \% \tag{S3}$$

The deviation of activity of each vial relative to the first vial $\Delta a_{\mathrm{rel},i}$ (%) was calculated using Eq. S4, where a_i and a_1 denote the activity concentration of vial i (Bq mL^{-1}) and vial 1 (Bq mL^{-1}).

$$\Delta a_{\text{rel},i} = \frac{a_i - a_1}{a_1} \times 100\ \% \tag{S4}$$

The relative recovered activity in leachate R_l (% of evaporated activity) was determined by Eq. S5. A_l and $A_\text{vap,ROH}$ represent the activity of the leachate and the evaporated alcohol, respectively (Bq). The activity of A_l was decay corrected to the measurement time of $A_\text{vap,ROH}$. The elapsed time interval between the two measurements is denoted as $t_\text{l,vap}$ (d).

$$R_\text{l} = \frac{A_\text{l}}{A_\text{vap,ROH}} \exp\left(\frac{\ln 2}{T_{1/2}} t_\text{l,vap}\right) \times 100\ \% \tag{S5}$$

Using the ratio of A_l and $A_\text{vap,ROH}$, the mass of evaporated alcohol $m_\text{vap,ROH}$ (mg) and mass of the zeolite in the beaker m_z (g), the mass of sorbed alcohol per mass of zeolite q (mg g^{-1}) was quantified by Eq. S6.

$$q = \left(\frac{m_\text{vap,ROH}}{m_\text{z}}\right) R_\text{l} \tag{S6}$$

The standard deviation of multiple measurements was used as the standard uncertainty of the mass of both the zeolite and the evaporated alcohol.

The uncertainties for each calculated physical property $U(y)$ was determined following Eq. S7. Here y represents the beforehand determined physical properties (a, A, $\Delta a_{\text{rel},i}$, R_l, q), $\frac{\partial y}{\partial x_i}$ the sensitivity coefficient and $u(x_i)$ the corresponding standard uncertainty for each individual input quantity.

$$U(y) = \sqrt{\sum \left(\frac{\partial y}{\partial x_i} \times u(x_i)\right)^2} \tag{S7}$$

References

(1) Feng, B.; Chen, B.; Zhao, C.; He, L.; Tang, F.; Zhuo, W. Application of a liquid scintillation system with 100-ml counting vials for environmental tritium determination: Procedure optimization, performance test, and uncertainty analysis. *J. Environ. Radioact.* **2020**, *225*, 106427. DOI: 10.1016/j.jenvrad.2020.106427.

(2) Chandran, K.; Nithya, R.; Sankaran, K.; Gopalan, A.; Ganesan, V. Synthesis and characterization of sodium alkoxides. *Bull. Mat. Sci.* **2006**, *29* (2), 173–179. DOI: 10.1007/BF02704612.

(3) Feng, B.; Ibesich, M.; Hainz, D.; Waidhofer, D.; Veit-Öller, M.; Trunner, C.; Stummer, T.; Foster, M.; Nemetz, M.; Welch, J. M.; et al. Development of a Novel Passive Monitoring Technique to Showcase the 3D Distribution of Tritiated Water (HTO) Vapor in Indoor Air of a Nuclear Facility. *Environ. Sci. Technol.* **2023**, *57* (48), 20024–20033. DOI: 10.1021/acs.est.3c05783.

(4) MacMahon, D. Half-life evaluations for ^{3}H, ^{90}Sr, and ^{90}Y. *Appl. Radiat. Isot.* **2006**, *64* (10), 1417–1419. DOI: 10.1016/j.apradiso.2006.02.072.
